# Acute Stroke Presenting as Unilateral Lower Extremity Monochorea

**DOI:** 10.7759/cureus.16116

**Published:** 2021-07-02

**Authors:** Dane O'Donnell, Mansoor Siddiqui

**Affiliations:** 1 Emergency Medicine, Thomas Jefferson University Hospital, Philadelphia, USA; 2 Emergency Medicine, Henry Ford Health System, Detroit, USA

**Keywords:** stroke, monochorea, mri, emergency medicine, pain, weakness

## Abstract

Stroke is a common condition that can present with a wide range of signs and symptoms based on the region of the brain experiencing impaired perfusion. As the diagnosis and treatment of acute strokes is frequently initiated in the emergency department (ED), early recognition by emergency providers is essential in providing patients with the best chance of recovery and symptom resolution while attempting to minimize the risk of disability. While lateralizing weakness, numbness, and speech changes are among the most common symptoms of a stroke, in rare cases, patients may present with subtle or atypical symptoms, necessitating a high index of suspicion as well as a thorough history and physical examination. We present a case of acute ischemic stroke presenting with isolated choreiform movement.

## Introduction

Stroke is a common condition that can present with a wide range of signs and symptoms based on the region of the brain experiencing impaired perfusion. As the diagnosis and treatment of acute strokes are frequently initiated in the emergency department (ED), early recognition by emergency providers is essential in providing patients with the best chance of recovery and symptom resolution while attempting to minimize the risk of disability. While lateralizing weakness, numbness, speech, and behavior changes are among the most common symptoms of a stroke, in rare cases, patients may present with subtle or atypical symptoms, necessitating a high index of suspicion as well as a thorough history and physical examination. We present a case of acute ischemic stroke presenting with isolated choreiform movement. 

## Case presentation

An 87-year-old Mandarin-speaking male with a past medical history of hypertension presented to the ED via an ambulance with a chief complaint of right lower extremity pain. He reported a history of difficulty ambulating worsening over the past week with frequent falls but could not recall injuring his right leg. Examination of his right lower extremity was notable for a poorly demarcated area of erythema and swelling. He also exhibited uncontrollable, writhing movements of the affected leg. His neurological examination was otherwise unremarkable. He demonstrated appropriate strength graded as 5/5 as well as intact sensation in bilateral upper and lower extremities. His reflexes were noted to be 2+ and symmetric in his bilateral lower extremities. His speech seemed uninhibited and a telephone interpreter did not remark on any difficulty in translation. 

**Figure 1:**
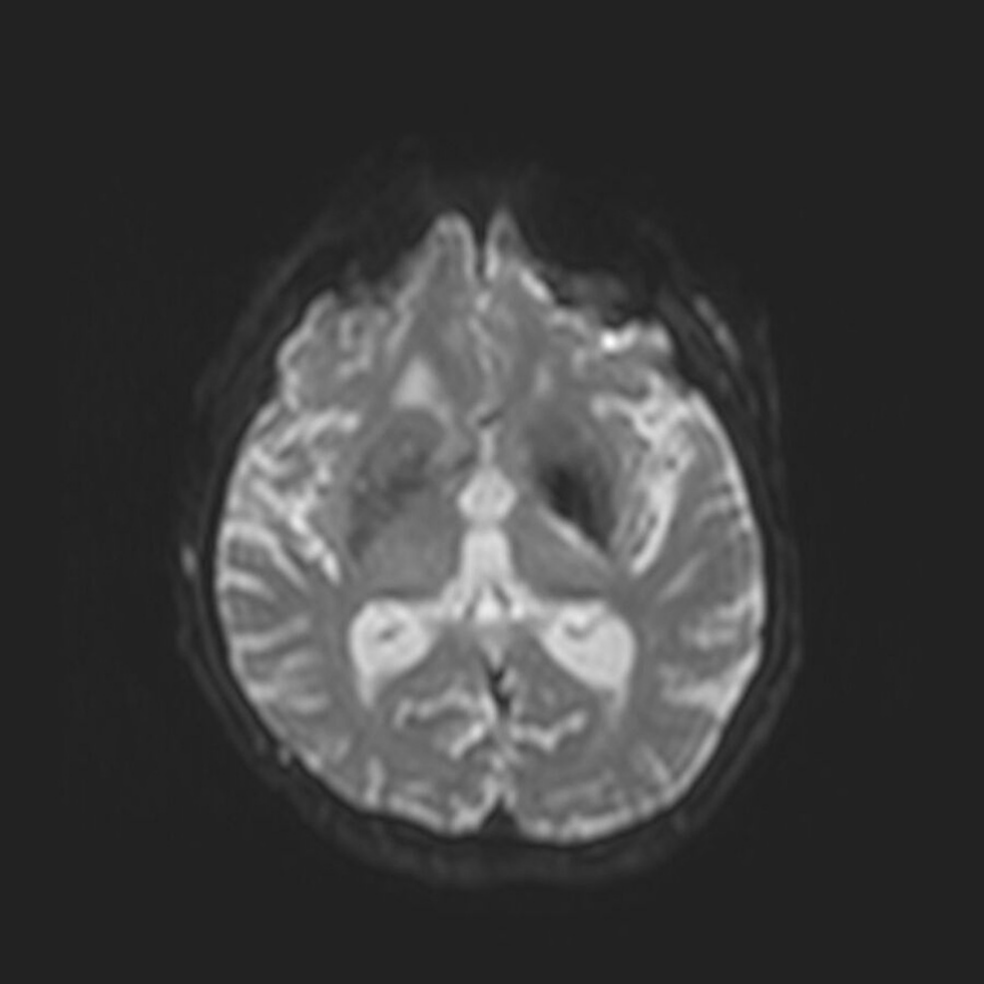
Diffusion weighted imaging showing restricted diffusion in the posterior limb of the left internal capsule.

**Figure 2:**
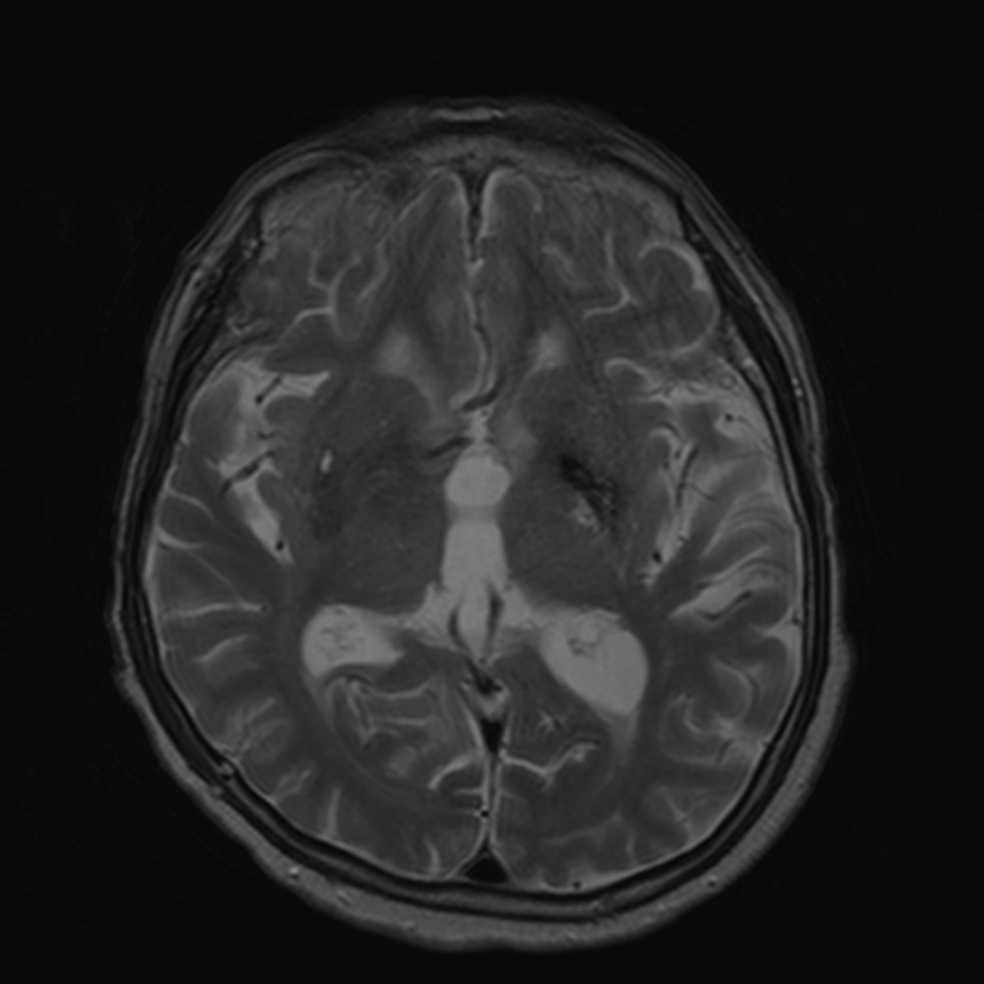
T2 weighted MRI showing evidence of prior hemorrhage in the left basal ganglia.

After consultation with the neurology service, MRI (Figures 1-2) was performed which showed acute infarction in the left posterior limb of the internal capsule as well as evidence of prior hemorrhage in the left basal ganglia. The patient was admitted to the stroke unit where he received risperidone 0.5 mg orally daily for the choreoathetoid movements which ameliorated his symptoms. He underwent physical therapy and was discharged to an acute rehab facility four days later.

## Discussion

Isolated movement disorders represent a rare manifestation of stroke. A search in PubMed revealed few case reports of strokes presenting with isolated monochorea, none of which were published in emergency medicine journals. Pandey and Gupta describe a similar case to ours of a 47-year-old male with sudden onset choreiform movement of the left lower extremity [1]. This patient also notably exhibited normal strength in the affected extremity. Kim et al. noted a similar case of a 67-year-old female who experienced symptoms for 20 days. Alike our patient, she received a dopamine antagonist (haloperidol) to ameliorate her symptoms [2]. Ito et al. describe a case in a 71-year-old female who had been experiencing 15 days of symptoms. Their article notes the rarity of developing choreoathetosis in the acute phase of a stroke [3].

Several challenges existed that could have impeded the ability of an emergency provider to recognize the patient’s symptoms as a potential consequence of a stroke including a language barrier, a non-neurologic chief complaint, and physical exam finding of lower extremity erythema and swelling that acted as a “red herring” versus the real cause of his presentation. Of note, the cause of the patient’s lower extremity erythema was not found, with an inpatient workup that included a negative lower extremity deep venous thrombosis study, and his symptoms improved throughout the patient’s hospitalization. It should also be noted that, upon initial evaluation, the patient did not exhibit any lower extremity weakness or change in sensation, symptoms that are most commonly associated with strokes. It is important to note that the basal ganglia hemorrhage was more likely the cause of the lower extremity monochorea rather than the acute stroke in the posterior limb of the internal capsule.

## Conclusions

Overall, this case reinforces the importance of obtaining a careful history and performing a thorough physical examination on patients presenting to the ED, especially in those with unusual symptoms. It also serves as a reminder to keep a high index of suspicion for ischemic cerebrovascular pathology in patients with new movement disorders. Specifically, the presence of new onset monochorea should prompt a workup for stroke, especially in patients with risk factors for cerebrovascular disease.
